# HIV-1 Subtype C-Infected Children with Exceptional Neutralization Breadth Exhibit Polyclonal Responses Targeting Known Epitopes

**DOI:** 10.1128/JVI.00878-18

**Published:** 2018-08-16

**Authors:** Zanele Ditse, Maximilian Muenchhoff, Emily Adland, Pieter Jooste, Philip Goulder, Penny L. Moore, Lynn Morris

**Affiliations:** a Centre for HIV and STIs, National Institute for Communicable Diseases of the National Health Laboratory Service (NHLS), Johannesburg, South Africa; b University of the Witwatersrand, Johannesburg, South Africa; c Department of Paediatrics and HIV Pathogenesis Programme, University of Oxford, Oxford, United Kingdom; d HIV Pathogenesis Programme, The Doris Duke Medical Research Institute, University of KwaZulu-Natal, Durban, South Africa; e Max von Pettenkofer Institute, Department of Virology, Ludwig Maximilian University Munich, Munich, Germany; f German Center for Infection Research (DZIF), partner site Munich, Germany; g Pediatric Department, Kimberley Hospital, Northern Cape, South Africa; h Centre for the AIDS Programme of Research in South Africa (CAPRISA), University of KwaZulu-Natal, Durban, South Africa; Emory University

**Keywords:** epitope mapping, HIV envelope targets, HIV vaccines, HIV-1 subtype C, mother-child transmission, neutralization breadth, neutralizing antibodies, pediatric immunology, bNAbs

## Abstract

We have previously shown that HIV-1-infected children develop broader and more potent neutralizing antibody responses than adults. This study aimed to determine the antibody specificities in 16 HIV-1 subtype C-infected children who displayed exceptional neutralization breadth on a 22-multisubtype virus panel. All children were antiretroviral treatment (ART) naive with normal CD4 counts despite being infected for a median of 10.1 years with high viral loads. The specificity of broadly neutralizing antibodies (bNAbs) was determined using epitope-ablating mutants, chimeric constructs, and depletion or inhibition of activity with peptides and glycoproteins. We found that bNAbs in children largely targeted previously defined epitopes, including the V2-glycan, V3-glycan, CD4bs, and gp120-gp41 interface. Remarkably, 63% of children had antibodies targeting 2 or 3 and, in one case, 4 of these bNAb epitopes. Longitudinal analysis of plasma from a mother-child pair over 9 years showed that while they both had similar neutralization profiles, the antibody specificities differed. The mother developed antibodies targeting the V2-glycan and CD4bs, whereas bNAb specificities in the child could not be mapped until 6 years, when a minor V2-glycan response appeared. The child also developed high-titer membrane-proximal external region (MPER) binding antibodies not seen in the mother, although these were not a major bNAb specificity. Overall, exceptional neutralization breadth in this group of children may be the result of extended exposure to high antigenic load in the context of an intact immune system, which allowed for the activation of multiple B cell lineages and the generation of polyclonal responses targeting several bNAb epitopes.

**IMPORTANCE** An HIV vaccine is likely to require bNAbs, which have been shown to prevent HIV acquisition in nonhuman primates. Recent evidence suggests that HIV-infected children are inherently better at generating bNAbs than adults. Here, we show that exceptional neutralization breadth in a group of viremic HIV-1 subtype C-infected children was due to the presence of polyclonal bNAb responses. These bNAbs targeted multiple epitopes on the HIV envelope glycoprotein previously defined in adult infection, suggesting that the immature immune system recognizes HIV antigens similarly. Since elicitation of a polyclonal bNAb response is the basis of next-generation HIV envelope vaccines, further studies of how bNAb lineages are stimulated in children is warranted. Furthermore, our findings suggest that children may respond particularly well to vaccines designed to elicit antibodies to multiple bNAb epitopes.

## Abstract (precis)

An HIV vaccine is likely to require bNAbs, which have been shown to prevent HIV acquisition in nonhuman primates. Recent evidence suggests that HIV-infected children are inherently better at generating bNAbs than adults. Here, we show that exceptional neutralization breadth in a group of viremic HIV-1 subtype C-infected children was due to the presence of polyclonal bNAb responses. These bNAbs targeted multiple epitopes on the HIV envelope glycoprotein previously defined in adult infection, suggesting that the immature immune system recognizes HIV antigens similarly. Since elicitation of a polyclonal bNAb response is the basis of next-generation HIV envelope vaccines, further studies of how bNAb lineages are stimulated in children is warranted. Furthermore, our findings suggest that children may respond particularly well to vaccines designed to elicit antibodies to multiple bNAb epitopes.

## INTRODUCTION

Fundamental differences exist between adult and pediatric HIV infection as a consequence of different transmission routes and immaturity of the pediatric immune system (1). The most significant difference is that HIV disease progression is more rapid in children and is associated with significantly higher levels of viral replication (2). Pediatric HIV infection is biphasic, with approximately 60% of deaths occurring before 3 years. Those children that survive beyond this usually show slow disease progression (3). About 10% of these children have normal CD4 counts in the context of high viral burden, which is only rarely seen in adult viremic nonprogressors (4). It was further shown that these pediatric nonprogressors (PNPs) had reduced CCR5 expression on long-lived memory CD4 T cells and low levels of immune activation (4). Protective HLA alleles play a less important role in nonprogressing HIV-1 infection in children, in contrast to adults (5). Furthermore, in adults, nonpathogenesis is associated with a high CD4 count and undetectable viral loads, whereas PNPs typically retain a persistently high viral burden (1, 4, 6). Previous studies have shown that neutralizing antibody responses in HIV-infected children arise early in the course of infection and are more potent and more broadly neutralizing than in adult infection (4, 7). These differences between adults and children provide a rationale for further characterizing humoral antibody responses during pediatric HIV-1 infection.

Broadly neutralizing anti-HIV antibodies (bNAbs) are considered an important component of an HIV vaccine and may also play a critical future role in treatment and cure strategies (8). Numerous studies have documented the presence of bNAbs in chronically infected adults (9–13). This has led to the isolation of a large number of broad and potent neutralizing monoclonal antibodies (MAbs) that have helped to define major sites of vulnerability on the HIV envelope, namely, the V2-glycan, V3-glycan, CD4 binding site (CD4bs), gp120-gp41 interface, and the membrane-proximal external region (MPER) (14). These targets provide a blueprint for immunogen design, although to date no envelope vaccines have been able to elicit bNAbs.

A previous longitudinal study of bNAbs in children has suggested that they arise earlier than in adults, and in some cases, bNAbs could be detected within the first year of life (7). Limited mapping data suggested that breadth was mediated either by multiple bNAb specificities that could not be deconvoluted or by bNAbs with novel specificities (7). Subsequently, a MAb was isolated and characterized from one infant (15). This bNAb, BF520.1, was shown to target the V3-glycan epitope on the HIV envelope and exhibited neutralization breadth of 58%. In addition, BF520.1 was shown to have low levels of somatic hypermutation compared with adult bNAbs targeting the same epitope, suggesting that the mechanisms governing development of bNAbs in children are different from those of adults.

We therefore sought to define bNAb specificities in a previously reported cohort of HIV-1 subtype C-infected children over 5 years of age who had high viral loads but normal CD4 counts and exceptionally high levels of bNAbs (4). Indeed, 70% (61/87) of children had plasma antibodies that neutralized ≥50% of viruses compared with only 19% of adults infected for a similar duration (4). In this study, we performed experimental mapping of bNAb specificities in 16 of these children. We showed that children develop bNAbs targeting epitopes similar to those of adults. However, we also demonstrated that the majority of children develop a polyclonal bNAb response targeting as many as four different epitopes. Lastly, we demonstrated that an infected mother-child pair developed equivalently high bNAb responses but that these target distinct neutralization epitopes, highlighting the role of multiple factors in bNAb development.

## RESULTS

### Clinical features of HIV-1 subtype C-infected children selected for this study.

**FIG 1:**
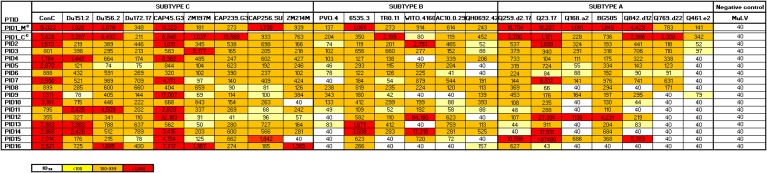
Breadth of plasma neutralizing activity from 16 children and 1 transmitting mother. Plasma samples were tested against 22 heterologous viruses from subtypes A, B, and C using the TZM-bl neutralization assay. Neutralization titers for each virus-plasma combination are shown as the reciprocal of the plasma dilution required to inhibit 50% virus infection (ID_50_). Data are shown as a heat map, with titers of >1,000 in red, those between 100 and 999 in orange, and those of <100 in light yellow, as indicated by the key. Titers of 1:40 indicate no neutralization. MuLV was included as a negative control. These data were used to derive the GMT included in Table 1.

We selected 16 HIV-1-infected children from two pediatric cohorts of nonprogressors and progressors plus one transmitting mother for epitope mapping based on their neutralization breadth and potency, as described in an earlier study (4). Clinical and demographic features of selected participants are shown in Table 1. The median age of the children was 10.1 years (interquartile range, 6.5 to 12.9), and all were antiretroviral treatment (ART) naive at the time of sample collection. The viral load ranged between 1,500 and 490,000 copies/ml, and the absolute CD4 count ranged between 300 and ∼2,000 cells/mm^3^. This unusual phenotype of normal CD4 counts in a context of high viral burden was previously described for the larger cohort from which these children were selected (4). Neutralization breadth and potency of all 17 plasma samples were assessed against a multisubtype panel of 22 heterologous viruses (Fig. 1). Neutralization breadth ranged between 60 and 100%, and the geometric mean titers (GMT) ranged between 151 and 1,204 (Table 1). Three children plus the transmitting mother neutralized 100% of the subtype A, B, and C viruses. Although there was a significant correlation between neutralization breadth and viral load in the larger cohort (4), significance between these two variables was not maintained in this smaller selected group of broad neutralizers.

**TABLE 1:** Demographic and clinical features of 17 HIV-1-infected participants selected for epitope mapping

Participant ID	Age (yr)	Classification	Absolute CD4 (/mm^3^)	Viral load (copies/ml)	% Neutralization breadth^*a*^	GMT^*b*^
PID1_M^*c*^	35.5	Nonprogressor	509	22,000	100	1,204
PID1_C^*c*^	6.5	Nonprogressor	1,553	13,938	100	1,009
PID2	12.9	Nonprogressor	521	490,000	100	328
PID3	10.2	Nonprogressor	756	110,000	100	325
PID4	11.9	Nonprogressor	635	42,000	91	297
PID5	9.6	Nonprogressor	1,622	10,321	91	188
PID6	10.1	Nonprogressor	1,395	50,000	91	151
PID7	10.0	Progressor	327	22,467	91	386
PID8	11.6	Nonprogressor	1,115	6,430	86	197
PID9	8.6	Nonprogressor	1,120	58,400	86	181
PID10	10.7	Nonprogressor	1,330	11,900	82	194
PID11	10.1	Nonprogressor	717	140,000	82	166
PID12	10.5	Progressor	301	71,000	82	343
PID13	6.7	Progressor	334	387,134	82	268
PID14	9.6	Nonprogressor	1,877	4,574	73	450
PID15	11.1	Nonprogressor	1,310	1,585	73	296
PID16	11.5	Nonprogressor	1,032	99,000	60	196

a Neutralization breadth was measured against a multiclade panel of 22 viruses.

b GMT, geometric mean titer.

c Mother-child pair.

### Differential effect of gp120 adsorption on neutralization capacity of plasma suggests polyclonal bNAb responses.

**FIG 2:**
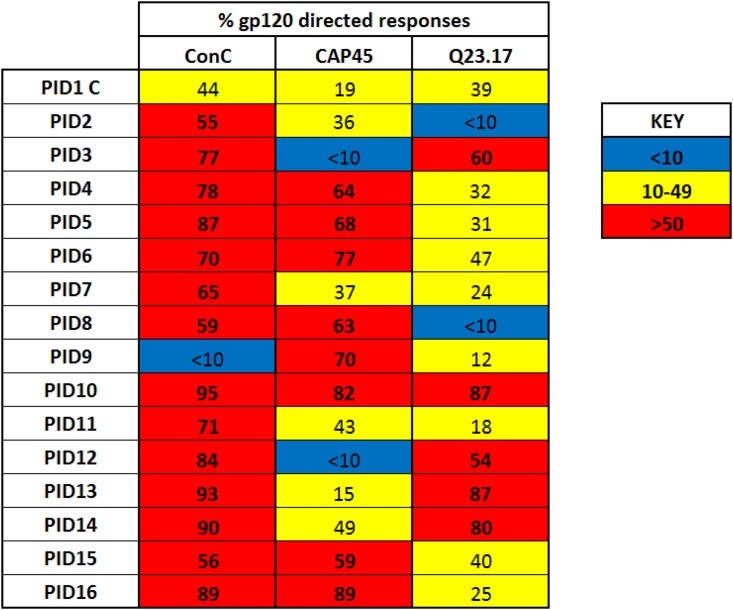
Differential effect of gp120 adsorption on neutralization suggests polyclonality. Anti-gp120 antibodies were depleted from plasma samples of all 16 children using tosyl-activated beads coated with ConC gp120 protein. Adsorbed plasma were tested for neutralization activity against ConC and heterologous viruses CAP45 and Q23.17. The percentage of gp120-directed neutralization was calculated as the reduction in ID_50_ of plasma incubated with gp120-coated beads relative to that of blank beads [((blank beads − gp120-coated beads)/blank beads) × 100]. Plasma samples where >50% of neutralizing activity was depleted by gp120 are colored red, and those with no depletion are colored blue. Those with some gp120-directed antibodies are colored yellow.

To assess whether gp120-directed antibodies mediated neutralization breadth, plasma samples were adsorbed with ConC gp120 and tested against ConC, CAP45, and Q23.17 in a TZM-bl neutralization assay. Enzyme-linked immunosorbent assays (ELISAs) confirmed the depletion of ConC gp120-binding antibodies for all plasma samples (data not shown). Adsorption with ConC gp120 resulted in at least a 50% reduction in neutralization activity for 14/16 plasma samples when tested against ConC, 8/16 samples for CAP45, and 5/16 samples for Q23.17 (Fig. 2). For one child (PID1_C), adsorption did not result in significant depletion of neutralization activity (>50%) against any of the 3 viruses, suggesting that neutralization breadth in this child is mediated by trimer-specific or gp41-directed antibodies. Interestingly, the effect of adsorption of gp120-specific antibodies on neutralization varied per virus for most plasma samples. For example, in PID12, gp120-directed antibodies neutralized ConC, partially neutralized Q23.17, but failed to neutralize CAP45, whereas in PID10 virtually all the neutralization activity against ConC, CAP45, and Q23.17 was gp120 directed (Fig. 2). This variable effect of gp120 adsorption by virus suggested that in most cases, breadth was mediated by polyclonal responses, some of which were gp120 specific.

### The majority of children develop antibodies targeting the V2-glycan and V3-glycan epitopes.

**FIG 3:**
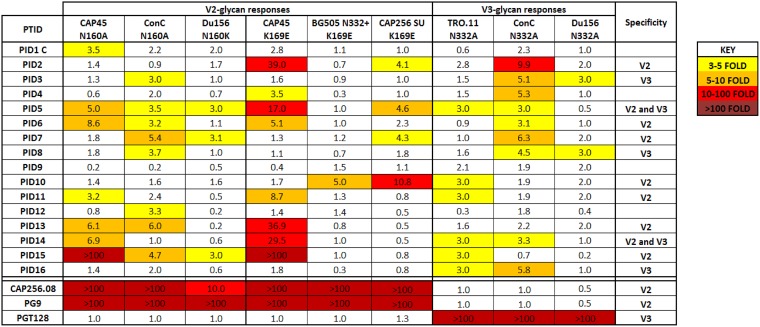
Majority of children develop neutralizing antibodies directed at V2-glycan and V3-glycan epitopes. Plasma samples from 16 children were assessed for V2-glycan antibodies using N160A/K and K169E mutants and for V3-glycan-directed responses using N332A mutants. Three heterologous viruses were assessed for each mutation and results are color coded with 3- to 5-fold reduction in neutralization activity shown in yellow, 5- to 10-fold reduction in orange, 10- to 100-fold reduction in red, and >100-fold reduction in dark red. V2/V3 specificity was assigned when plasma showed ≥3-fold reduction in at least 2 backbones. The V2 MAbs CAP256.08 and PG9 and the V3 MAb PGT128 were used as positive controls and showed the expected activity.

Pseudoviruses containing mutations at key residues in the V2-glycan and V3-glycan epitopes were used to screen for these specificities in plasma samples. For V2-glycan antibodies, we used both N160A/K and K169E mutants, as some V2-directed antibodies, such as the CAP256-VRC26 lineage, exhibit limited glycan dependence (16, 17). Specificity was assigned when neutralizing activity for the mutant virus was ≥3-fold less than that of the wild-type virus in 2 or more backbones. By these criteria, 9 of the 16 children (56%) developed antibodies directed at the V2-glycan epitope (Fig. 3), although the effect varied by virus backbone. For example, in PID15, either a 160A or 169E mutation completely abrogated neutralization in CAP45, suggesting that the V2 specificity accounts for all activity against this virus. However, neither BG505 nor CAP256_SU was neutralized by V2-directed antibodies, suggesting that other specificities in this child were responsible for neutralizing these viruses. Similarly, a >10-fold effect was observed for PIDs 2, 5, 13, and 14 in CAP45, but the effect was less pronounced for other backbones. Several plasma samples were more affected by the K169E mutation than by the N160A mutation in CAP45, suggesting less dependence on the glycan moiety in this epitope, similar to CAP256 MAbs (16, 17). Consistent with published data, neutralization by positive-control MAbs CAP256.08 and PG9 was reduced/knocked out in V2-glycan mutants (16–18).

To assess V3-glycan antibodies, we deleted the N332 glycan in three backbones, TRO.11, ConC, and Du156.12. Using the same cutoffs, a V3-glycan specificity was assigned to a third (5/16, 31%) of the children, of whom four (PIDs 2, 5, 7, and 13) also showed evidence of V2-glycan-directed bNAbs (Fig. 3). Consistent with previous data (19), we observed a knockout in neutralization activity for PGT128 against the N332A mutant in all viruses tested. Overall, our data suggest that bNAbs directed at the V2-glycan epitope are the most common in HIV-1 subtype C-infected children with broad neutralizing responses.

### CD4bs-directed plasma antibodies are frequently detected in children.

**FIG 4:**
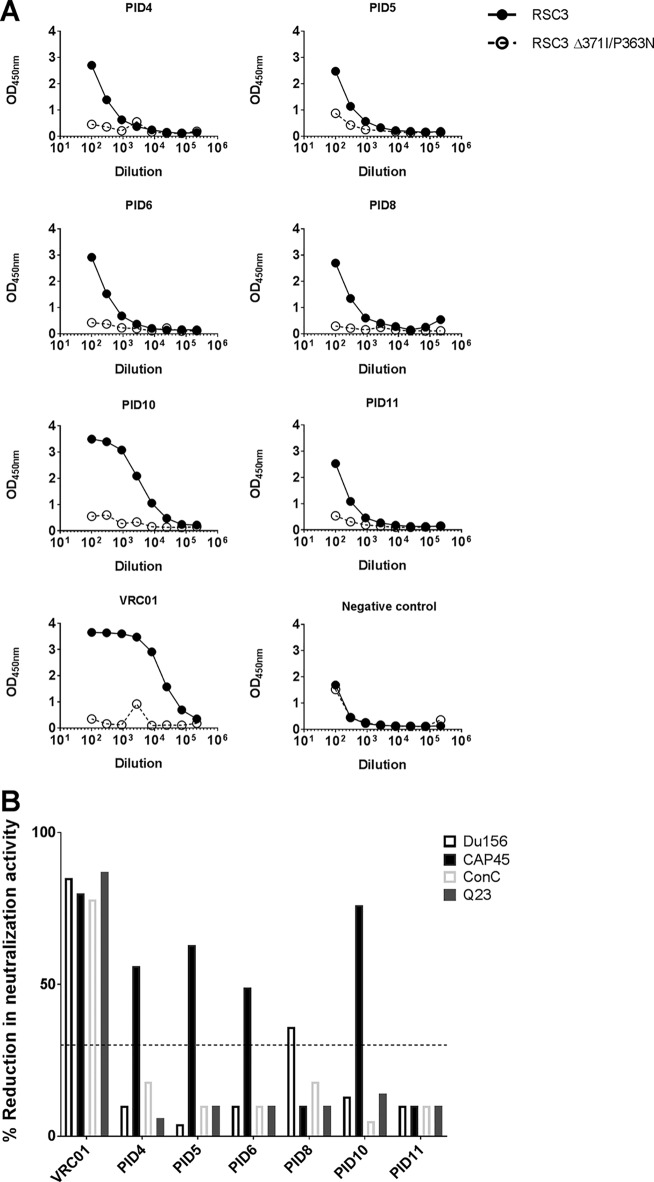
Mapping of CD4bs antibodies by binding and neutralization using the RSC3 protein. (A) Plasma samples were tested for binding to the RSC3 protein (solid line) and the CD4bs mutant protein RSC3Δ371I/P363N (dotted line) by ELISA. Shown are curves of 6 children with binding to the RSC3 protein but not the mutant protein, similar to VRC01 and suggestive of CD4bs antibodies. PID14 showed equivalent binding to both proteins and was used as a negative control. (B) The RSC3 protein was also used in a competition neutralization assay to detect CD4bs antibodies in the same 6 participants. Neutralization of Du156.12, CAP45, ConC, and Q23.17 was measured following incubation with RSC3. A reduction in neutralization of ≥30% (dotted line) was considered positive. Five of the 6 children showed activity against one virus, most commonly CAP45. VRC01 was used as a positive control, with RSC3 blocking neutralization of all 4 viruses as expected.

We used the wild-type RSC3 protein and its RSC3Δ371I/P363N mutant to assess CD4bs binding and neutralization activity. Binding of six plasma samples (PIDs 4, 5, 6, 8, 10, and 11) to the RSC3Δ371I/P363N CD4bs mutant was significantly lower than that to the wild-type RSC3 protein, suggesting the presence of CD4bs-directed antibodies in these participants (Fig. 4). This pattern was comparable to that of the positive-control MAb, VRC01 (Fig. 4), which was isolated using this RSC3 probe. Overall, the binding assays demonstrated that a third of children (6/16, 38%) had CD4bs-directed activity. Protein competition assays using the RSC3 protein verified CD4bs-directed neutralization activity in all six children (Fig. 4B). This was confirmed by at least 30% reduction in neutralization activity following incubation with RSC3 protein. This effect was, however, only observed in one virus for 5 of the 6 children (Fig. 4B). Incubation with the RSC3Δ371I/P363N CD4bs mutant did not impact neutralization activity against any of the 4 viruses tested (data not shown).

In addition to binding assays, neutralization assays were performed using viruses with mutations in the CD4bs epitope (N276A, N279A, and R456W). The N276 and N279 residues are important contact sites for HJ16-like CD4bs antibodies, while the R456 residue is critical for epitope recognition for VRC01, 3BNC117, N6, and VRC27 (20). No reduction in neutralization activity was observed with the N276A and N279A mutants for any participants (data not shown). However, a 4-fold reduction in neutralization activity using the R456W mutant was observed in two participants (PID4 and PID10) (data not shown), verifying the data using the RSC3 proteins. Overall, binding and neutralization assays suggest that ∼40% of children develop CD4bs-directed antibody responses that contribute to broad neutralization activity.

### Anti-MPER antibodies rarely confer broad neutralizing activity in children.

**FIG 5:**
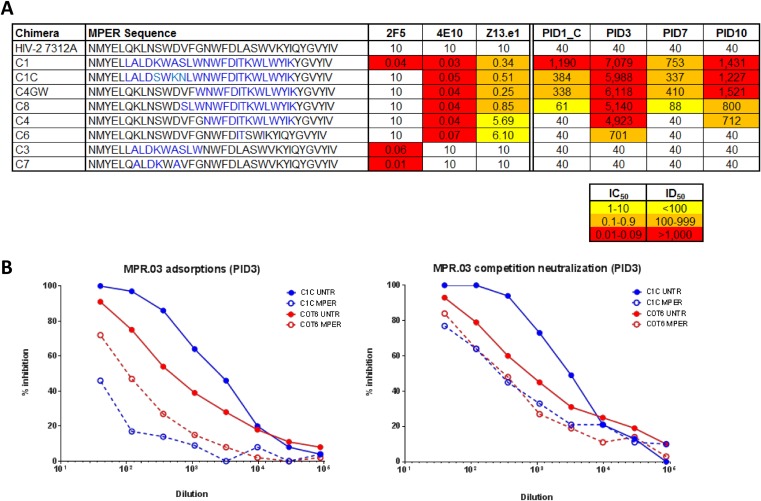
Mapping of anti-MPER neutralizing activity in four children. (A) Plasma samples from 4 children with high levels of neutralizing activity against the HIV-2/HIV-1 MPER C1 and C1C constructs were tested against 6 additional chimeric constructs containing point mutations to map known MPER antibodies. Also shown are the sequences carried by the MPER of each engrafted HIV-2/HIV-1 chimeric construct. Mutated amino acids are indicated in blue. Titers are shown as ID_50_ and are color coded with HIV-2 used as a negative control. 2F5, 4E10, and Z13e1 were used as positive controls. (B) Plasma from PID3 was incubated either with magnetic beads coated with MPR.03 peptide (left) or incubated directly with the MPR.03 peptide (right) to adsorb anti-MPER antibodies. MPER broad neutralization activity against untreated (solid lines) and adsorbed (dotted lines) samples was tested against heterologous viruses C1C and COT6. Adsorption of anti-MPER antibodies in PID3 reduced neutralization of heterologous viruses.

The presence of MPER-directed antibodies in plasma samples was measured using the HIV-2/HIV-1 MPER chimeras as previously described (21). Four of 16 children showed 50% reciprocal plasma dilution (ID_50_) titers of >750 against either C1 (contains the subtype B HIV-1 MPER sequence) or C1C (subtype C HIV-1 MPER sequence). Fine mapping of these 4 samples was performed using additional chimeras, C4, C4GW, C6, and C8, that contain point mutations in the MPER (21). PID3 and, to some extent, PID10 had a 4E10-like footprint characterized by potent neutralization of C4GW, C4, C8, and C6 (Fig. 5A). Two samples, PID1_C and PID7, had similar patterns, neutralizing C4GW and C8 but not C4 and C6, a footprint similar to that of Z13e.1 (Fig. 5A). None of the four plasma samples showed neutralization of C3 and C7 (which contain the 2F5 epitope), suggesting that recognition of this N-terminal MPER region is uncommon. These data indicate that the anti-MPER antibodies in children recognized an epitope within the C terminus of the MPER, similar to bNAb 4E10.

To assess the contribution of the MPER-directed antibodies to broad neutralization activity, adsorption and competition neutralization assays were performed using the MPR.03 peptide. PID3, who had the highest titers against the HIV-2/HIV-1 MPER chimeras, showed a significant reduction in neutralization activity in both assays. This effect was seen against C1C and COT6 (Fig. 5B) but not in another 3 heterologous viruses (data not shown). In contrast, no reduction in neutralization activity was observed for PID1_C, PID7, and PID10 against any of the viruses tested (data not shown). Overall, MPER antibodies were therefore shown to contribute to breadth in only 1/16 children in this cohort.

### Antibodies directed to the gp120-gp41 interface detected in more than half of the children.

**FIG 6:**
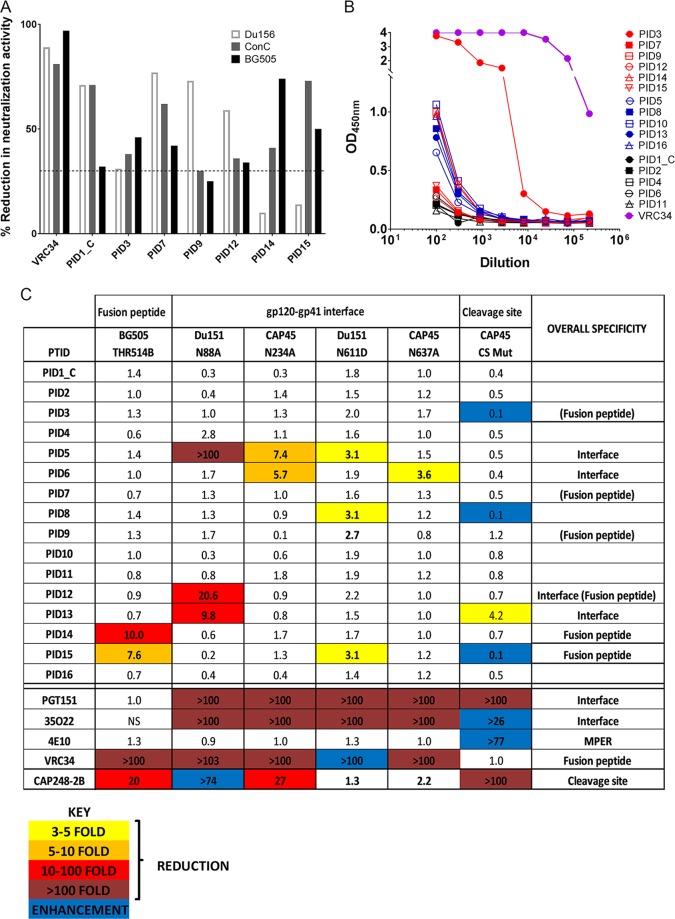
Children develop antibody responses to the gp120-gp41 interface, including the fusion peptide. (A) A reduction in neutralization of Du156.12, ConC, and BG505 was measured following incubation with the fusion peptide FP9. A cutoff of ≥30% (dotted line) was considered significant. Of the 16 children, 7 showed activity against 2 or 3 viruses that was FP directed. VRC34 was used as a positive control. (B) Plasma samples from 16 children were assessed for binding to FP9 using ELISA. Samples where FP9 competed for neutralization in panel A are shown in red, blue represents ELISA-positive samples where FP9 did not compete for neutralization, and black represents samples that were negative for both binding and neutralization. VRC34 was used as a positive control. (C) Mapping of responses using mutant viruses to the fusion peptide (THR514B), gp120-gp41 (residues 88, 234, 611, and 637), and the cleavage site (CAP45 CS Mut that contains 6 mutations known to expose the MPER). The fold change in neutralization breadth for the mutants relative to the wild-type strains is shown as a heat map for all 16 plasma samples. Loss of activity is shown in yellow (3- to 5-fold), orange (5- to 10-fold), and red (>10-fold), and enhancement is shown in blue. PGT151, 35O22, 4E10, VRC34, and CAP248-2B were used as positive controls. The overall specificity is shown in the last column, with those in brackets derived from data shown in panels A and B.

To determine whether children have antibodies directed at the gp120-gp41 interface, including the fusion peptide (FP), plasma samples were screened using peptide competition assays, ELISAs, and neutralization assays with epitope-ablating mutants. The FP9 peptide inhibited >30% neutralization against at least 2 of the 3 viruses (Du156.12, ConC, and BG505) in 7 children, suggesting the presence of fusion peptide-directed antibodies (Fig. 6A). As described by Kong et al., we also observed >80% reduction in neutralization activity against all 3 viruses for VRC34, a fusion peptide-specific antibody used as a positive control (22) (Fig. 6A). ELISA data demonstrated binding responses to the FP9 peptide for 11 children (Fig. 6B), six of whom also exhibited evidence of FP-mediated competition (Fig. 6B, red curves). The remaining five ELISA-positive children did not show inhibition of neutralization (Fig. 6B, shown in blue). Interestingly, no binding antibodies to FP9 were observed for PID1_C, although FP9-specific neutralizing responses were detected by competition (Fig. 6A and B).

To further assess FP-directed responses in children, we also used a viral mutant containing a threonine insertion after residue 514 in the FP of isolate BG505. This mutation confers complete resistance to VRC34 (22). A 10- and 8-fold decrease in neutralization activity against this mutant strain was observed for PID14 and PID15 (Fig. 6C), both of whom also had FP responses by ELISA and competition neutralization assays (Fig. 6A and B), suggesting that the fusion peptide is a target for bNAbs in these two children.

Since gp120-gp41 interface bNAbs 8ANC195, PGT151, 35O22, VRC34, and CAP248-2B target distinct but overlapping epitopes (23), we tested mutants critical for each bNAb. Three children had antibodies sensitive to deletion of the glycan at position 88 in gp120, with fold effects from 10 to >100, one of whom, PID12, was also shown to have FP bNAbs. The N88 glycan is critical for epitope recognition for PGT151, 35O22, and VRC34 (23) (Fig. 6C). We also observed sensitivity to the N88A and N234A mutations for PID5 and for PID6 against the N234A mutation only, as has been reported for 8ANC195-like antibodies (14) (Fig. 6C). Three plasma samples, including PID5, showed a minor effect with N611D, and PID6 neutralization was reduced by N637A; both mutations affect PGT151, 35022, and VRC34 neutralization.

We next screened plasma samples using the CAP45 CS-Mut virus that contains 6 mutations in the gp120-gp41 cleavage site, which we described previously (23). These mutations mediate escape from PGT151 and CAP248-2B-like antibodies but result in exposure of the MPER detected through increased neutralization sensitivity (23). As previously reported, CAP45 CS-Mut was >100-fold more resistant to PGT151 but showed enhancement of 35022 and 4E10 (Fig. 6C). A 4-fold reduction in neutralization activity was observed against CAP45 CS-Mut in one child, PID13, suggesting that PGT151/CAP248-like antibodies contribute to neutralization activity in this participant (Fig. 6C). We saw enhanced neutralization activity in 3 participants (PID3, PID8, and PID15), suggesting these children had MPER-directed or 35O22-like antibodies (Fig. 6C). In support of this, we observed MPER activity in PID3 (Fig. 4A and B) and, to some extent, PID8 (C1C titer of 188; data not shown). Overall, our data demonstrate that more than half of children (9/16, 56%) develop antibodies targeting regions within the gp120-gp41 interface.

### Neutralization breadth in children is mediated by multiple antibody specificities.

**FIG 7:**
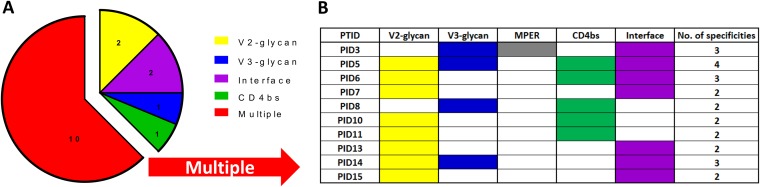
The majority of children develop antibodies targeting multiple epitopes. (A) Pie chart showing a summary of mapped neutralization epitopes targeted by plasma antibodies from children. (B) Breakdown of specificities detected in children with more than one bNAb. Shown in the last column is the total number of mapped specificities.

Combining all the mapping data generated in this study, we noted that the majority of children (10/16, 63%) developed more than one antibody specificity (Fig. 7A). Most had 2 specificities, although 3 children had three specificities and one child, PID5, developed antibodies targeting four different epitopes (Fig. 7B). None of the children developed bNAbs that targeted the MPER epitope only. No association was observed between neutralization breadth and the number of bNAb specificities detected in each child's plasma sample.

### Mother-child pair develops antibodies targeting distinct bNAb epitopes.

**FIG 8:**
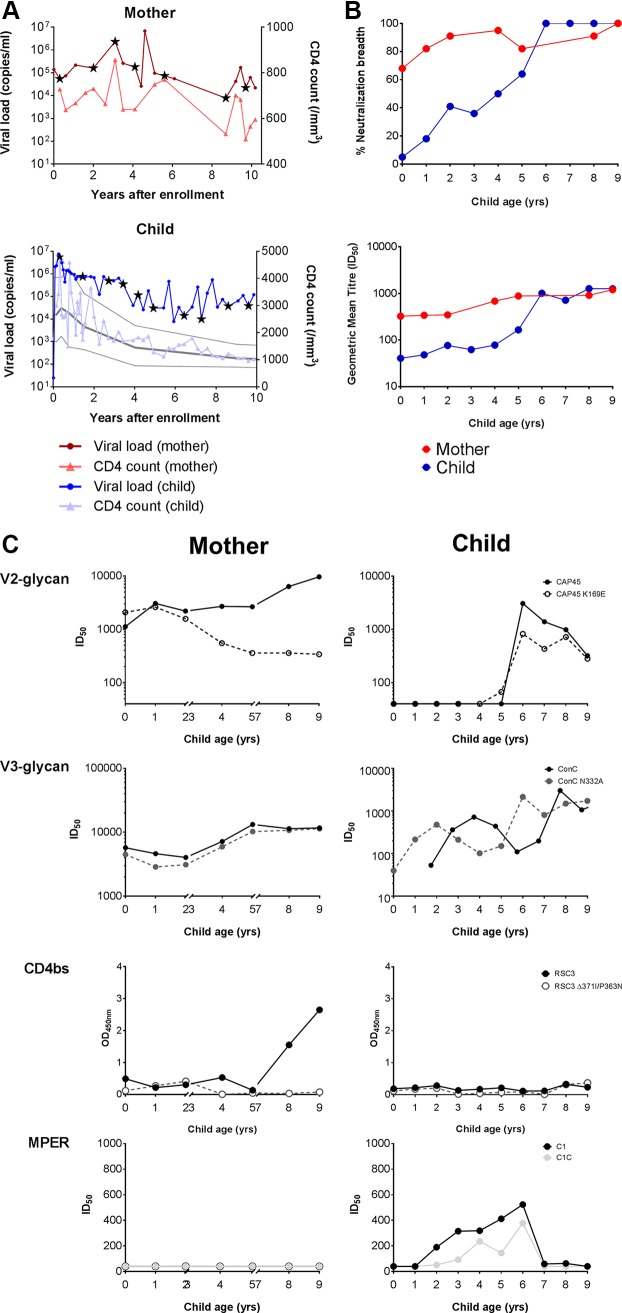
Mother-child pair develop antibodies that target distinct epitopes. (A) Longitudinal viral load (circles) and CD4 counts (triangles) for mother-child pair. Mother's data (PID1_M) are shown in red, and the child's data (PID1_C) are shown in blue. Stars indicate the time points used for plasma mapping. Absolute CD4 counts with the median and 10th and 90th percentiles for normal uninfected children over the first 10 years of life are shown in gray (53, 54). (B, top) Kinetics of development of neutralization breadth in the mother-child pair over a 9-year period. Shown is the percentage of viruses neutralized in a 22-virus panel with mother shown in red and child in blue. (Bottom) Longitudinal geometric mean ID_50_ titers (GMT) of all viruses in the 22-virus panel for mother and child. (C) Mapping of the specificities over time in mother and child. Longitudinal samples were assessed for V2-glycan using the K169E mutant in CAP45 relative to the wild-type strain. V3-glycan-directed responses were assessed using the N332A mutant in ConC relative to the wild-type strain. CD4bs activity was assessed by the RSC3 and the RSC3Δ371I/P363N CD4bs mutant in ELISA. MPER responses were determined using the MPER chimeric constructs C1 (black) and C1C (gray). Wild-type strains are shown in solid lines and closed circles, and mutants are shown in dotted lines. Mother's data (PID1_M) are shown on the left, and the child's data (PID1_C) are on the right. Mother's samples were missing at time points 3, 6, and 7 years of the child's age.

To further understand factors associated with development of neutralization breadth, we studied a mother-child pair over a 9-year period. The mother (PID1_M) was enrolled into the study approximately 8 years after infection and had a viral load of 139,000 that fluctuated over time but remained high (Fig. 8A). Her CD4 count remained above 500 cells/ml. The child's (PID1_C) viral load was greater than 2 million copies/ml at birth and declined slowly over time but was above 100,000 copies/ml at 9 years. At enrollment into this study the mother's plasma neutralized 60% of viruses in the 22-virus panel tested, rising to 100% over the next 9 years (Fig. 8B). In contrast, no neutralizing activity was observed in the child at 3 months, but breadth emerged over time and by 6 years of age onwards the child's plasma also neutralized 100% of the viruses tested (Fig. 8B). Geometric mean titers were >1,000 for both mother and child after more than 9 years of infection.

We had initially mapped the 6- and 9-year postenrollment time point for the child and mother, respectively. To examine the evolution of bNAb responses in the mother-child pair we mapped the longitudinal samples (Fig. 8C). Comparison of CAP45 and CAP45 K169E titers showed the development of a V2-glycan response from 3 years onwards in the mother. This effect was verified in 2 different backbones (CAP45 and BG505 [data not shown]). A slight V2 dependence was observed for the child at 6 and 7 years, suggesting that V2-glycan bNAbs were not a major specificity in the child. We observed no V3 responses in either mother or child, as there were no differences between the N332A mutant relative to the wild-type strain. CD4bs activity was seen in the mother at later time points; however, this specificity was not evident in the child. The child developed MPER binding antibodies from 2 years of age, which persisted until 6 years of age and then declined over time. No MPER activity was observed in the mother. Overall, our data suggest that the mother-child pair developed different antibody profiles, with antibodies directed at the V2-glycan and CD4bs epitopes evolving in the mother while the major specificities mediating breadth in the child could not be mapped.

## DISCUSSION

Although many studies have examined the development and targets of bNAbs in adults (14, 24–28), a limited number of studies have assessed these responses in children following HIV-1 infection (4, 7, 15). We and others have shown that children mount broader and more potent antibody responses than adults, which indicates fundamental differences in the way children respond to HIV antigens (4, 7). In the present study, we used several approaches to reveal the targets of bNAbs among a large number of preselected HIV-1 subtype C-infected children. We observed that although neutralizing antibody specificities were similar to those of adults, the majority of children had bNAbs targeting multiple epitopes. This polyclonality may be due to the long duration of infection and high viral loads in this group of children, features strongly linked to the development of neutralization breadth in adults (10, 11, 24, 25, 29). These data support the notion that children mount stronger and more complex responses to HIV antigens than adults, which may have implications for the immunogenicity of HIV vaccines in children.

Polyclonal bNAb responses in adults have been previously reported (10, 19, 30–32); however, this occurs at a much lower frequency than that observed here in children, where 63% had between 2 and 4 specificities. Our previous data from the CAPRISA cohort suggested that of the 7 adult women who developed bNAbs, only 1 had more than one specificity (33). Similarly, longitudinal studies from the protocol C adult cohort demonstrated multiple bNAb specificities in only 3/42 (7%) participants (10). However, this may be an underrepresentation, since many adult mapping studies are performed earlier in infection than with the children in this study.

Examples of adults with multiple specificities following long-term infection have been reported, such as the single donor from whom the antibodies 35O22 and 10E8 were isolated after 20 years of infection (34, 35). Furthermore, the transmitting mother in this study was shown to have at least 2 bNAb responses. In this case, she was a highly unusual viremic nonprogressor who maintained normal CD4 counts for over 17 years and a median viral load of 85,000 copies/ml (Fig. 8). Collectively, these data suggest that long duration of persistent exposure to high antigenic load, which is more common in children, can facilitate high levels of bNAb activity targeting multiple neutralization epitopes.

Previous reports have shown that bNAbs arise more rapidly in children and can be detected within a year of infection (7). This was also seen in the longitudinal sampling of PID1_C, where some neutralizing activity was detected by 1 year. Over the next 6 years this increased to 100% neutralization of the panel, remaining at these levels for another 3 years. In adults, neutralization breadth generally takes up to 3 years to emerge, although cases of earlier detection of neutralizing activity have been reported (18, 25, 36). Further studies are needed to determine whether children have unique immunologic features that shorten the normally long process associated with the development of bNAbs. The isolation of MAb BF520.1 from an infant targeting the V3-glycan epitope on the HIV envelope, which exhibited low levels of somatic hypermutation and few indels, supports this possibility (15). Breadth in children may also be the result of their ability to generate new B cell lineages for a longer period of time, whereas data in adults suggests new bNAb specificities rarely emerge after 4 to 5 years of infection (25, 29).

The majority of children had responses directed at the V2-glycan epitope. This is similar to findings in adults (10, 19, 25, 32), suggesting that this antigenic site on the HIV-1 envelope is dominant in adult and pediatric HIV infection. The high proportion of CD4bs responses in this cohort may also be a consequence of the long duration of infection, as this is a prerequisite for this class of antibodies, which requires the accumulation of high levels of somatic hypermutation over many years (20, 37). However, isolation and genetic characterization of CD4bs MAbs from children is needed to address this question. Binding responses to the fusion peptide were observed in the majority of children, similar to a subtype B adult cohort, where 42% of the participants had this specificity (22). In contrast, MPER responses were rarely found in children, which is similar to the case for adults (13, 31, 38, 39). Structural constraints in accessing the MPER on gp41 as well as the autoreactivity of MPER-targeting antibodies are thought to be some of the factors that contribute to the restricted induction of responses against this site (40, 41). PID3 had the highest titers of MPER bNAbs and showed enhanced neutralization of the CS mutant, which has an exposed MPER region (23). In addition, high titers of antibodies against the fusion peptide in PID3 further supports the conclusion that gp41 was exposed on the virus that infected this child.

The presence of multiple antibody specificities did not correlate with neutralization breadth, as exemplified by PID5, who developed 4 distinct bNAb specificities but did not neutralize all viruses in the panel. Despite the array of techniques used here, neutralization breadth in some children could not be completely mapped, suggesting the possibility that bNAbs with novel specificities were also present, as has been reported in adult cohorts. In particular, breadth in PID1_C could not be clearly mapped, and the lack of adsorption with gp120 suggested that the bNAbs were trimer specific or targeted gp41. Isolation of MAbs from this child would therefore be of interest.

Mother-to-child transmission provides a unique setting to examine the role of viral and host factors that shape bNAb responses. This study demonstrated that the mother-child pair developed bNAbs targeting distinct neutralization epitopes. The high degree of viral and host genetic relatedness suggests that multiple factors shape the development of bNAbs. There was no neutralizing activity in the child at 3 months of age, indicating that responses in the child arose *de novo* and that maternal bNAbs, if present, had waned to below detectable levels. The bottleneck in HIV mother-to-child transmission is well described, with children often becoming infected with a minor variant (42, 43). Antibody specificities in both mother and child evolved continuously, and studies are ongoing to examine viral evolution and escape pathways in this pair.

Most studies aimed at HIV vaccine development have been conducted in adults. However, studies in infants have demonstrated that HIV vaccination induces strong immune responses (44, 45). Importantly, Fouda et al. reported robust and durable anti-V1V2 IgG antibody responses to MF59-adjuvanted HIV vaccines in HIV-exposed infants compared with those of RV144 recipient adults (46). These differences in vaccine responses between adults and infants were shown to be attributable to modulation of gp120-directed responses by adjuvants (47). Our finding that children develop broader and more potent responses to HIV infection suggests that they respond better to vaccines than adults.

In summary, our study demonstrates that children who develop broad and potent antibodies target multiple specificities directed at previously identified epitopes. This suggests that children are fundamentally better at generating bNAb responses than adults (1, 4, 6, 7, 46, 47). It will be important to understand how these responses are induced during infection and whether this is due to the unique immunological environment of the immature immune system that allows multiple B cell lineages to thrive. Isolation and characterization of MAbs from children will also help to address this. Importantly, our data suggest that children respond well to vaccine immunogens currently under clinical evaluation. Testing of bNAb-based HIV vaccines in children should therefore be a priority.

## MATERIALS AND METHODS

### Study population.

This study included ART-naive, HIV-1 subtype C-infected PNPs and progressors recruited from Kimberley and Durban, South Africa, as part of a study to determine the immunological factors associated with nonpathogenesis in children (4). The PNP cohort consists of ART-naive children with CD4 counts of >750 cells/mm^3^ and viral loads of >14,000 copies/ml who have not met the clinical criteria for ART initiation. The progressor cohort consists of ART-naive children whose absolute CD4 count had declined to <500 cells/mm^3^ and had viral loads of >70,000; these children are at the current threshold of ART initiation (4) according to national guidelines for ART initiation at the time when the participants were recruited. For this study, plasma samples collected in EDTA from 16 children with the highest neutralization breadth were selected for epitope mapping. For child PID1_C (also known as 517-C), samples from the transmitting mother were also tested. Ethical approval was obtained from the University of the Free State Ethics Committee, University of KwaZulu-Natal Biomedical Research Ethics Committee, and University of Oxford Research Ethics Committee (4).

### Adsorption of anti-gp120 antibodies.

Anti-gp120 antibodies were depleted using monomeric wild-type ConC gp120 covalently coupled to tosyl-activated magnetic beads as described previously (31, 48, 49). Adsorbed plasma samples were assessed in ELISAs to confirm depletion of anti-gp120 antibodies and in the TZM-bl neutralization assay against HIV-1 to assess residual activity (31, 48, 49).

### Neutralization assays.

Neutralization was measured as the reduction in luciferase gene expression following a single round of infection of TZM-bl cells with Env-pseudotyped viruses (50). Titers were calculated as the 50% inhibitory concentration (IC_50_) or the reciprocal plasma dilution (ID_50_) causing a 50% reduction of relative light units (RLU) compared to results for the virus-treated or untreated control wells (50). Mutations were made in the following backbones: TRO.11, ConC, Du156.12, CAP45.G3, Du151.2, BG505 N332+, and CAP256 SU. BG505 N332+ is a BG505 strain where a glycan at position 332 was knocked in, and CAP256 SU is the superinfecting virus from CAPRISA participant CAP256. For neutralization competition assays, peptides or proteins were added to each well at a concentration of 250 μg/ml. This included the synthetic biotinylated fusion peptide, FP9-biotin, with the sequence AVGIGAVFL (22), the MPR.03 peptide with the sequence KKKNEQELLELDKWASLWNWFDITNWLWYIRKKK (51), and the resurfaced stabilized gp120 core (RSC3) and its CD4bs mutant, RSC3Δ3711/P363N (52). Proteins/peptides were incubated with serially diluted MAbs or human plasma for 30 min at 37°C before addition of pseudovirus. The mixture was further incubated for 30 min at 37°C. TZM-bl cells were added at 0.5 million cells/ml and incubated for 48 h, followed by cell lysis and measurement of luciferase activity.

### Detection of antibodies targeting the MPER.

Anti-MPER activity was measured using chimeric HIV-2/HIV-1 MPER constructs (51). Plasma responses of an ID_50_ of >750 against either C1 or C1C were considered positive, and these were further investigated using adsorption experiments. Adsorption experiments were performed using streptavidin-coated magnetic beads (Dynal MyOne Streptavidin C1; Invitrogen) incubated with biotinylated peptides carrying the MPR.03 sequence as described previously (51). Adsorbed plasma samples were tested by ELISA to confirm depletion of anti-MPER antibodies and in TZM-bl neutralization assays to assess the contribution of MPER antibodies to breadth (51).

### ELISA.

MPER, RSC3, and fusion peptide ELISAs were performed as described previously (21, 22, 52). Briefly, 96-well ELISA plates (Corning, USA) were coated with 2 μg/ml of protein/peptide overnight. Coated plates were washed with phosphate-buffered saline (PBS) containing 0.05% Tween (dilution buffer) and blocked with 5% goat serum, 5% skim milk in dilution buffer for 1 h at 37°C. A 100-fold dilution of each plasma sample was added to the wells, titrated, and incubated for 1 h at 37°C. Unbound antibodies were removed by 4 washes before addition of goat anti-human IgG peroxidase-conjugated (KPL) at a 1:1,000 dilution. Following incubation with the secondary antibody, the wells were washed four times and developed using 1-Step Ultra TMB substrate (Thermo Scientific, Waltham, MA, USA). The reaction was stopped with 0.2 M H_2_SO_4_, and adsorbance was read at an optical density of 450 nm on a microplate reader (Molecular Devices).
